# Managing Placenta Accreta and Massive Hemorrhage: A Case Report on Anesthetic and Surgical Interventions

**DOI:** 10.7759/cureus.64071

**Published:** 2024-07-08

**Authors:** Marta Laranjo, Leonor Aniceto, Catia Domingues, Luís Gonçalves, João Fonseca

**Affiliations:** 1 Anaesthesiology, Unidade Local de Saúde da Região de Leiria, Leiria, PRT

**Keywords:** perioperative management, lifesaving hysterectomy, massive haemorrhage, placenta accreta, obstetric haemorrhage

## Abstract

Obstetric haemorrhage is a leading cause of maternal morbidity and mortality and is a common reason for intensive care unit (ICU) admission in the postpartum. Primary postpartum obstetric haemorrhage is associated with four main causes: tone, thrombin, trauma, and tissue. Regarding the last one, placenta accreta is an abnormal invasion of the placenta into the myometrium. Early diagnosis of placenta accreta allows for better perioperative management; however, it is sometimes only identified during caesarean delivery when the placenta cannot be removed. We report a case of a 37-year-old woman with a history of caesarean section due to placenta previa, who was admitted at 36 weeks and 1 day for an urgent caesarean section (c-section) due to cord presentation. A subarachnoid block (SAB) was used for anaesthesia. It was chosen over general anaesthesia because it allows the patient to experience the birth of her children, enhances pain control, and avoids complications associated with general anaesthesia. Besides our centre has expertise in neuraxial anaesthesia. During the procedure, placental accretism and massive haemorrhage occurred, and a life-saving abdominal hysterectomy was needed. The patient experienced hypotension, partially responsive to volume replacement and vasopressors, leading to norepinephrine infusion and conversion to general anaesthesia. The surgery lasted 2.5 hours with a blood loss of 3500 ml. The patient was extubated without complications and transferred to the post anaesthesia care unit (PACU). Risk factors for placenta accreta spectrum (PAS) include previous surgery and placenta previa with a prior c-section. Antenatal diagnosis is crucial, and women with risk factors should undergo imaging at experienced centres. Delivery centres must have protocols for unexpected PAS and major obstetric haemorrhage. Both general and neuraxial anaesthesia can be suitable for managing PAS, and caesarean hysterectomy is often required to control haemorrhage. Postoperatively, adequate monitoring and care is essential. PAS management should involve excellent communication between a multidisciplinary team in specialised centres.

## Introduction

Obstetric haemorrhage is an important cause of maternal morbidity and mortality and the main obstetric cause of admission to the ICU. It´s classified into antepartum obstetric haemorrhage or postpartum obstetric haemorrhage (PPH) [1].

PPH can be primary or secondary. Primary PPH occurs within 24 hours postpartum. Secondary PPH occurs after 24 hours postpartum up to six weeks postpartum. Its aetiology is associated with the ‘four Ts’: tone (anomalies in uterine contraction), thrombin (anomalies in coagulation), trauma (injury to the genital area or uterine rupture) and tissue (retention of conception products or placenta accreta) [1]. Major obstetric haemorrhage is defined as cumulative blood loss equal to or greater than 1000 ml or blood loss accompanied by signs and symptoms of hypovolemia within 24 hours after birth [2].

Placenta accreta is defined as the pathological invasion of the uterine wall’s myometrium by the placenta and is part of a spectrum of pathologies involving invasion of the uterine wall that includes placenta increta, percreta, and accreta [3,4]. The maternal mortality rate, hysterectomy rate and hospital stay are higher for women with this pathology [4].

Placenta accreta occurs in 3% of women diagnosed with placenta previa without a previous caesarean and its incidence is increasing. Risk factors include previous caesarean section, number of previous caesareans, and placenta previa [4]. For women with placenta previa, the risk of placenta accreta is 3%, 11%, 40%, 61%, and 67% for the first, second, third, fourth, and fifth or more caesareans, respectively [4,5].

Ideally, the diagnosis of placenta accreta is established in the antenatal period so that the optimisation of perioperative management can be anticipated and planned. Sometimes it´s only recognised at the time of the caesarean when attempts to remove the placenta are unsuccessful [4].

## Case presentation

We present a 37-year-old woman with a history of caesarean section due to placenta previa. Currently pregnant at 36 weeks and 1 day, with a spontaneous dichorionic diamniotic twin pregnancy. No other significant medical history.

Upon admission to the emergency department, the cardiotocogram identified two foetal heart rates at 135 and 140 bpm with good variability. The obstetric ultrasound (US) revealed one foetus in cephalic presentation with cord presentation and the second foetus in transverse presentation. The laboratory results showed no abnormalities. She was admitted to the operating room for an urgent c-section in the context of a cord presentation and previous caesarean. A subarachnoid block (SAB) was chosen as an anaesthetic technique, using 12 mg of isobaric levobupivacaine and 3 mcg of sufentanil at L3-L4 intervertebral space, under standard monitoring, with two peripheral intravenous access and active body warming. After the SAB, a decrease in blood pressure required vasopressor support with boluses of 10 mg of ephedrine, up to 50 mg.

**Figure 1:**
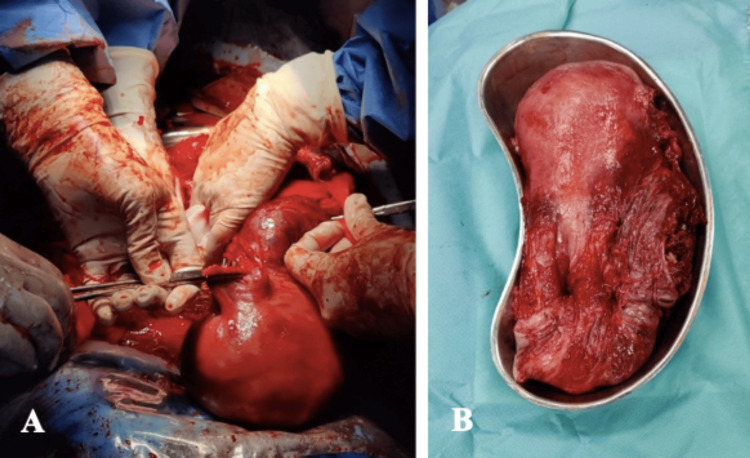
A) Uterus during the surgery. B) Anatomical specimen after hysterectomy.

Antibiotic prophylaxis was made with 2 g of cefazolin. Additionally, 8 mg of ondansetron, 40 mg of esomeprazole and 20 mg of metoclopramide were administered. The surgical procedure was initiated, with the extraction of the foetuses and APGAR (Appearance, Pulse, Grimace, Activity, Respiration) scores of 9/10 for both live births. Subsequently, placenta accreta with massive haemorrhage was identified, leading to the decision for a life-saving abdominal hysterectomy (Figure 1).

The hemodynamic profile became hypotensive, partially responding to volume replacement and 25 mg of ephedrine, prompting the administration of 50 to 100 mcg boluses of phenylephrine, totalling 350 mcg until norepinephrine infusion (200 mcg/ml) was initiated via a peripheral venous catheter at a rate of 5 ml/h, adjusted based on hemodynamic response. This was started 30 minutes after the beginning of the surgery and continued for an hour until adequate volume replacement. Meanwhile, a third larger bore intravenous access was established (16 G). A total volume of 1 l of warmed crystalloid and 1 l of gelatine were administered.

Massive haemorrhage protocol was activated and the following blood products were administered: five units of packed red blood cells, three units of fresh frozen plasma, one pool of platelets, 2 g of fibrinogen, and 1.5 g of tranexamic acid. Electrolyte imbalances, specifically hypocalcaemia, were corrected with 2 g of calcium chloride. Intraoperatively, two arterial blood gases were performed, showing good evolution (Table 1). An additional dose of 1 g of cefazolin was administered given the blood loss. Intraoperative antibiotic redosing is needed to ensure adequate serum and tissue concentrations of the antimicrobial if the duration of the procedure exceeds two half-lives of the drug or there is excessive blood loss (greater than 1500 ml) during the procedure [6].

**Table 1:** Intraoperative blood gas analyses

	Time Since Anaesthesia Beginning	
Intraoperative Blood Gas	1 h 40 min	3 h 05 min
pH	7.340	7.290
pCO_2_ (mmHg)	36.0	43.0
pO_2_ (mmHg)	349.0	295.0
Hg (g/dl)	3.7	9.1
Ht (%)	11.0	27
SpO_2_ (%)	100	99
Na‑ (mmol/l)	136	135
K^+^ (mmol/l)	3.9	4.0
Ca^2+^ (mmol/l)	1.30	1.11
Cl^-^ (mmol/l)	115	111
Glucose (mg/dl)	131	191
Lactate (mmol/l)	2.6	2.1
HCO_3_- (mmol/l)	19	21
Base excess (mmol/l)	-6	-6

To minimise haemorrhagic losses and until the bleeding site was controlled, the mean arterial pressure (MAP) target was 50 mmHg, which was compatible with the patient’s intact cognitive capacity. After haemorrhage control, the MAP target was set to >70 mmHg.

After 50 minutes of surgery, for patient comfort and better situational and hemodynamic control, the anaesthetic technique was converted to general anaesthesia. Induction was made with 50 mcg of fentanyl, 20 mg of etomidate, and 75 mg of succinylcholine. Orotracheal intubation with a video laryngoscope was performed without complications. Anaesthesia maintenance included 50 mg of rocuronium and 4% desflurane, monitoring anaesthetic depth with processed electroencephalogram. Mechanical ventilation was set in volume control mode with protective ventilation parameters and FiO_2_ at 60%.

**Figure 2:**
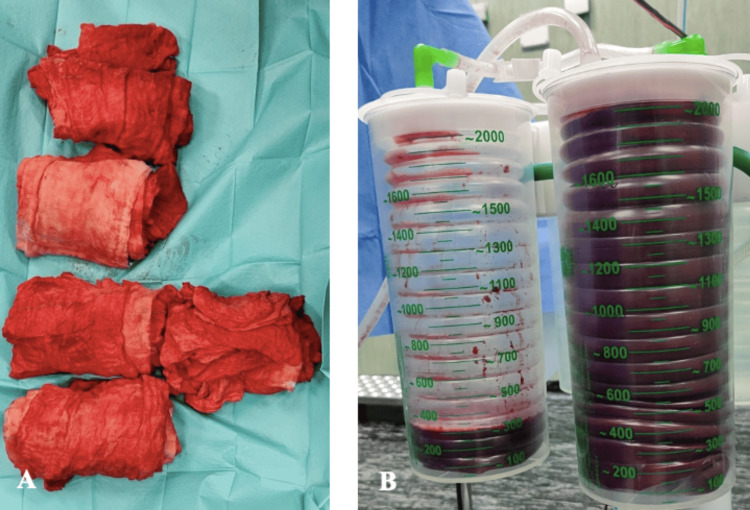
Measurable intraoperative blood loss. A) Blood in compress. B) Blood in a surgical suction reservoir.

The surgical procedure lasted about 2.5 hours, with an estimated blood loss of 3500 ml and a total urine output of 600 ml (Figure 2).

Before extubating, intravenous paracetamol was administered and a bilateral ultrasound-guided transversus abdominis plane block with 0.2% ropivacaine was performed, with 20 ml on each side.

After the complete reversal of the neuromuscular block, the patient was extubated in the operating room without complications and transferred to the PACU, where she spent the night under continuous monitoring until discharge to the ward the next day. In the immediate postoperative analytical study, hypoalbuminemia was noted, and 50 ml of 20% albumin was administered (Table 2).

**Table 2:** Analytical evolution from admission till the sixth postoperative day (PO).

		Admission	Immediate PO	Day 1	Day 2	Day 3	Day 5	Day 6
Hemogram	Hg (g/dl)	12.4	12.3	8.3	9.7	6.2	7.8	8.8
	Ht (%)	35.1	35.7	23.2	29.4	18	22.2	25.8
	VCM (fl)	96.7	90.3	88.9	72.7	91.8	89.6	91.9
	HCM (pg)	34.1	31.0	31.9	24.1	31.5	31.7	31.5
	RDW (%)	18.7	15.8	16.4	15.6	17.5	16.3	16.7
	PLT (10^3^/ul)	145	103	106	208	159	173	313
Coagulation	INR	0.95	1.09	1.05	-	-	-	-
	TP (seconds)	10.40	-	11.60	-	-	-	-
	aPTT (seconds)	26.0	26.0	26.6	-	-	-	-
Biochemical	Blood glucose (mg/dl)	122	-	-	-	-	-	-
	Urea (mg/dl)	22	-	-	-	-	-	-
	Creatinine (mg/dl)	0.55	-	-	-	-	-	-
	Na^+^ (mmol/l)	141	-	-	-	-	-	-
	K^+^ (mmol/l)	3.9	-	-	-	-	-	-
	Ca^2+^ (mmol/l)	2.18	-	-	-	-	-	-
	Mg^2+^ (mmol/l)	0.68	-	-	-	-	-	-
	Cl^-^ (mmol/l)	108	-	-	-	-	-	-
	Albumin (g/dl)	22	-	-	-	-	-	-

On the first postoperative day, blood analysis revealed hypomagnesaemia (Mg2+ 0.68 mmol/L), so 1 g of intravenous magnesium sulphate was given. Besides 100 mg of intravenous iron was started daily. On the third postoperative day, with a haemoglobin level of 6.2 g/dl, in collaboration with haematology, it was decided to administer two units of packed red blood cells, and the removal of the abdominal drain was postponed.

She was discharged on the seventh postoperative day, with oral iron prescribed and prophylactic enoxaparin, to complete 10 days of thromboembolic prophylaxis.

## Discussion

It’s crucial to stratify patients in order to plan for a safe delivery. The most important risk factors for PAS are previous surgery or endometrium manipulation and current placenta previa with previous c-section [5,7]. Our patient had important risk factors that should trigger an adequate timing for surgery, between 34 weeks and 35 weeks gestation in order to optimise neonatal outcomes and minimise maternal risk [4,5,7]. For optimal patient outcome and referral, antenatal diagnosis is important and women with risk factors should undergo imaging in centres with experience in US diagnosis of PAS. US and magnetic resonance imaging (MRI) have similar sensitivity and specificity to screen PAS and no synergistic benefit [5]. However, a US or MRI with negative findings does not exclude the presence of PAS. So, delivery centres should have protocols for unanticipated PAS and for major obstetric haemorrhage [5,7].

Regarding anaesthetic technique choice, both general and neuraxial anaesthesia may be appropriate for patients with PAS and each has its specific advantages. General anaesthesia provides comfort in long procedures, allows one to electively approach a possible difficult airway and in case of massive haemorrhage does not have the sympathectomy associated with neuraxial block [7]. On the other hand, the neuraxial approach enhances intra and postoperative pain control, avoids airway manipulation, allows an awake mother to experience childbirth and has minimal foetus exposure to anaesthetics [5,7]. These advantages of the neuraxial approach and the expertise of our centre with this technique lead to its choice in this case. Additionally, retrospective studies suggest that neuraxial anaesthesia can be safely performed even when a caesarean hysterectomy is anticipated or required, making it a suitable choice for managing the suspected PAS in this case [5]. A study reported that neuraxial anaesthesia was the preferred modality (95%) in women suspected of PAS undergoing non-emergent c-sections for placenta previa, and the conversion rate to general anaesthesia after delivery was 12% [8].

In this case, neuraxial anaesthesia was then converted to general anaesthesia to secure the airway, better manage massive haemorrhage, large volume resuscitation and vasopressor titration. It also facilitates abdominal wall relaxation and surgical exposure. It should be anticipated and treated hypotension from the combined effect of neuraxial sympathectomy and response to intravenous anaesthetics plus active haemorrhage [5].

In patients whose risk factors go unrecognised, unexpected PAS can only be identified intraoperatively [5]. The obstetric centres should be prepared for emergency management in these scenarios and massive haemorrhage protocols should exist according to the specificity of each institution, which typically releases a defined ratio (1:1:1) of platelets, fresh frozen plasma and packed red blood cells [2,4]. If blood loss exceeds 1,5 L, prophylactic antibiotics should be redosed, as shown in the reported case [4,6].

Goal-directed fluids and their warming reduce dilutional coagulopathy. We recognise that point-of-care coagulation testing can provide useful information about coagulation status and guide transfusion and may be associated with reduced blood product use [5,7], but in our hospital, these devices are not timely available. In a large randomised controlled trial, tranexamic acid one gram intravenously at the onset of postpartum haemorrhage decreased the risk of death, with no increase in thrombotic complications [9].

The most common surgical approach to PAS is caesarean hysterectomy with placenta left in situ after delivery of the foetus. Attempts of placental separation increase the risk of haemorrhage [4]. In this case, there was uncontrollable bleeding, so a hysterectomy was performed even though fertility preservation was originally planned.

Regarding postoperative care, patients should receive vigilant monitoring in the early postoperative period [4,5]. Frequently this is best done in the ICU [4,7]. Admission can be determined by the need for ongoing organic support or the anticipated need for further transfusion, to ensure hemodynamic and haemorrhagic stabilisation [4,5]. In this case, the patient made an immediate recovery in the PACU which could provide adequate monitoring and care.

As for pain control, a multimodal approach should be offered, with paracetamol, NSAIDs, neuraxial analgesia and/or truncal blocks, such as transversus abdominis plane block [5,7].

## Conclusions

This case highlights the critical importance of seamless communication among team members, timely and informed decision-making, and adherence to institutional massive haemorrhage protocols in managing obstetric emergencies such as placenta accreta. Effective coordination and immediate interventions significantly improve this patient outcome. Furthermore, debriefing after highly demanding situations is essential for continuous improvement, and meticulous postoperative care ensures patient recovery and reduces complications, and it’s an area for improvement in our institution. This case underscores the necessity for comprehensive, multidisciplinary approaches in complex obstetric cases to enhance maternal and neonatal safety.
